# Peptidomics dataset: Blood plasma and serum samples of healthy donors fractionated on a set of chromatography sorbents

**DOI:** 10.1016/j.dib.2018.04.018

**Published:** 2018-04-10

**Authors:** Georgij Arapidi, Maria Osetrova, Olga Ivanova, Ivan Butenko, Tatjana Saveleva, Polina Pavlovich, Nikolay Anikanov, Vadim Ivanov, Vadim Govorun

**Affiliations:** a Shemyakin-Ovchinnikov Institute of Bioorganic Chemistry of the Russian Academy of Sciences, Miklukho-Maklaya str. 16/10, Moscow 117997, Russian Federation; b Moscow Institute of Physics and Technology (State University), Institutskii Per. 9, Moscow Region, Dolgoprudny 141700, Russian Federation; c Federal Research and Clinical Center of Physical Chemical Medicine of the Federal Medical and Biological Agency of the Russian Federation, Malaya Pirogovskaya 1a, Moscow 119435, Russian Federation

## Abstract

Blood as connective tissue potentially contains evidence of all processes occurring within the organism, at least in trace amounts (Petricoin et al., 2006) [1]. Because of their small size, peptides penetrate cell membranes and epithelial barriers more freely than proteins. Among the peptides found in blood, there are both fragments of proteins secreted by various tissues and performing their function in plasma and receptor ligands: hormones, cytokines and mediators of cellular response (Anderson et al., 2002) [2]. In addition, in minor amounts, there are peptide disease markers (for example, oncomarkers) and even foreign peptides related to pathogenic organisms and infection agents. To propose an approach for detailed peptidome characterization, we carried out an LC–MS/MS analysis of blood serum and plasma samples taken from 20 healthy donors on a TripleTOF 5600+ mass-spectrometer. We prepared samples based on our previously developed method of peptide desorption from the surface of abundant blood plasma proteins followed by standard chromatographic steps (Ziganshin et al., 2011) [3]. The mass-spectrometry peptidomics data presented in this article have been deposited to the ProteomeXchange Consortium (Deutsch et al., 2017) [4] via the PRIDE partner repository with the dataset identifier PXD008141 and 10.6019/PXD008141.

**Specifications table**

**Table:** 

Subject area	*Biochemistry*
More specific subject area	*Proteomics, peptidomics*
Type of data	*LC–MS/MS data and identification data*
How data was acquired	*TripleTOF 5600+ mass spectrometer with a NanoSpray III ion source (Sciex, Canada) coupled with a NanoLC Ultra 2D+ nano-HPLC system (Eksigent, USA)*
Data format	*Raw and analyzed data*
Experimental factors	*Blood plasma and serum samples of 10 healthy male and 10 healthy female donors*
Experimental features	*Samples were fractionated on several sorbents (cation exchange Toyopearl CM-650M, CM Bio-Gel A, SP Sephadex C-25 and anion exchange QAE Sephadex A-25) and analyzed by LC–MS/MS individually and pooled*
Data source location	*Shemyakin-Ovchinnikov Institute of Bioorganic Chemistry of the Russian Academy of Sciences, Miklukho-Maklaya str. 16/10, Moscow 117997, Russian Federation*
Data accessibility	*The mass spectrometry peptidomics data have been deposited to the ProteomeXchange Consortium via the PRIDE*[5]*partner repository with the dataset identifier*PXD008141 and 10.6019/PXD008141. Direct download link: http://www.ebi.ac.uk/pride/archive/projects/PXD008141

**Value of data**

• The dataset contains 59 raw LC–MS/MS analyses of blood plasma and serum samples fractionated on several different chromatography sorbents. The chromatography methods used complement each other.
• The dataset contains a large number of identified peptides, fragments of known human proteins. The dataset describes the possibilities of our approach for detailed peptidome characterization.
• The dataset can be analyzed for novel peptides, e.g. products of possible lncRNA translation.
• The dataset allows for extended statistical analysis, and we encourage such collaborations.

## 1 Data

Blood plasma and serum samples of 10 healthy male and 10 healthy female donors were fractionated on a set of sorbents (cation exchange Toyopearl CM-650M, CM Bio-Gel A, SP Sephadex C-25 and anion exchange QAE Sephadex A-25) and analyzed by LC–MS/MS individually and pooled in equal quantities, separately for male and female samples (Table 1, Supplementary Table S1).

**Table 1:** Number of analyzed samples in each group.

Biomaterial type	Chromatography sorbent	Sample type	Number of samples
Plasma	Toyopearl	Individual	4 male, 4 female
		Pool	6 male, 3 female
Serum	Toyopearl	Individual	4 male, 4 female
		Pool	2 male, 2 female
	Bio-Gel	Pool	8 male, 7 female
	SP-Sephadex	Pool	2 male, 3 female
	QAE-Sephadex	Pool	6 male, 4 female

Initial analysis allowed to identify 13,590 unique peptides belonging to 1430 protein groups. The distribution of the identified peptides by groups is shown in Table 2 (more information can be found in Supplementary Table S2).

**Table 2:** Number of identified peptides and spectra.

Sample group	Number of identified peptides	Number of identified spectra
Plasma, Toyopearl	4516	100,730
Serum, Toyopearl	4207	78,304
Serum, Bio-Gel	5058	67,409
Serum, SP-Sephadex	1635	7407
Serum, QAE-Sephadex	3614	25,782
Total number	13,590	279,632

**Fig. 1:**
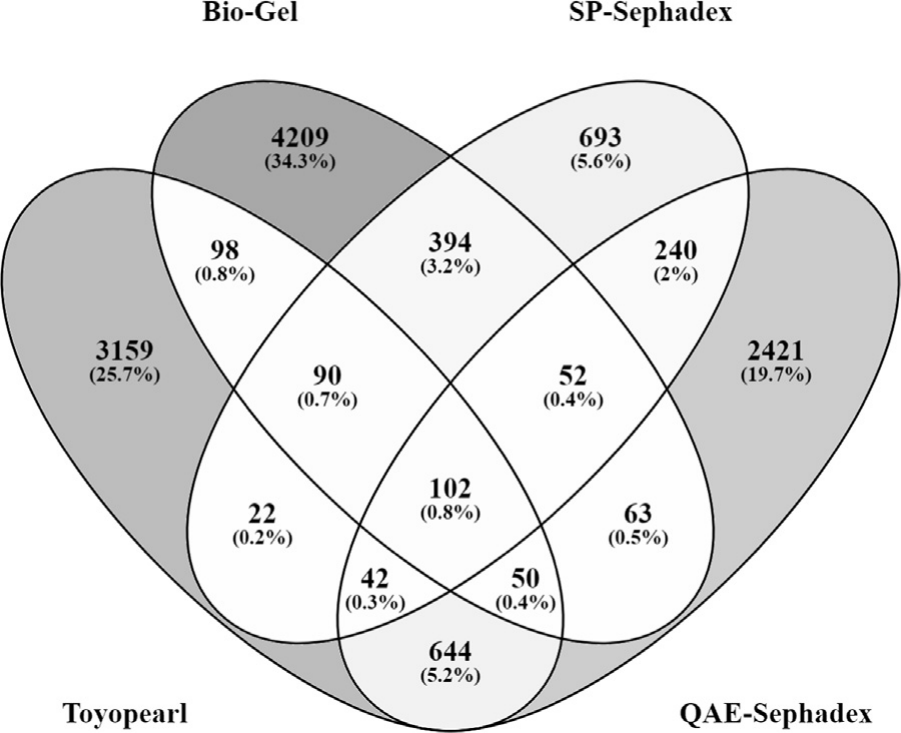
Number of common and unique peptides for blood serum sample groups.

**Fig. 2:**
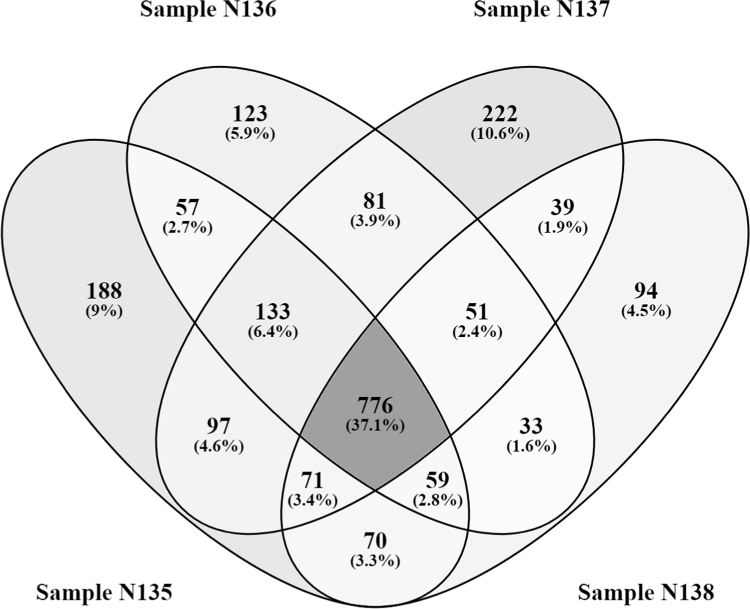
Number of common and unique peptides for 4 individual blood plasma samples.

The chromatography methods used complement each other since the identified peptides are fairly unique for a particular sample group (Fig. 1). On the other hand, the identified peptides are sufficiently reproducible within the sample group – approximately 70% of the peptides are reproduced in at least two out of four samples (Fig. 2).

**Fig. 3:**
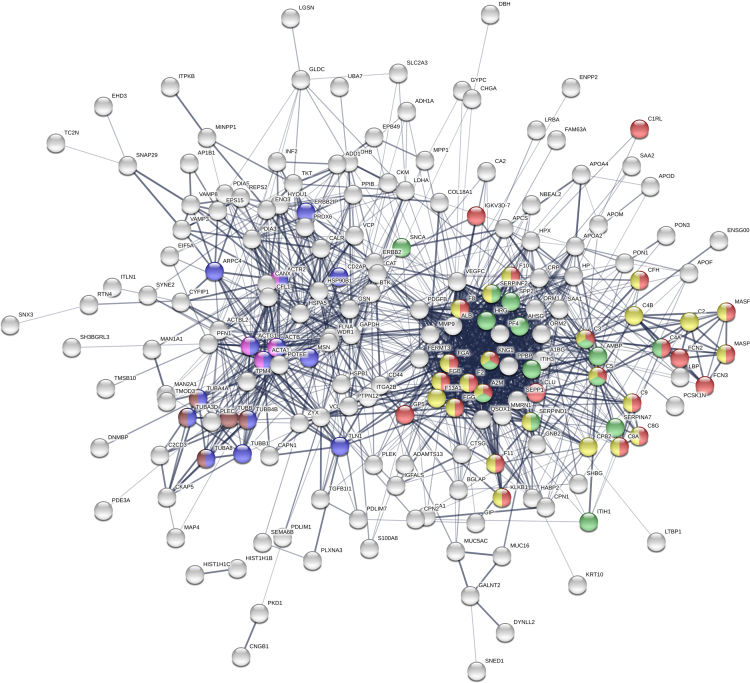
Protein–protein interaction network of the precursor proteins of the identified peptides. The analysis via STRING database. Red - “protein activation cascade” pathway; Green - “endopeptidase inhibitor activity” pathway; Yellow - “Complement and coagulation cascades” pathway; Blue - “structural constituent of cytoskeleton” pathway; Purple - “Actin” pathway; Brown - “Tubulin C-terminal domain” pathway.

The analysed known protein-protein interactions between precursor proteins of the identified peptides via STRING (Search Tool for the Retrieval of Interacting Genes/Proteins) database [6] revealed two perfectly seen clusters (Fig. 3). Functional enrichment analysis shown that one cluster consists of proteins belong to pathways “protein activation cascade” (GO:0072376), “endopeptidase inhibitor activity” (GO:0004866) and “Complement and coagulation cascades” (KEGG:04610), while the other includes proteins of pathways “structural constituent of cytoskeleton” (GO:0005200), “Actin” (PF00022) and “Tubulin C-terminal domain” (PF03953). Most of the precursor proteins of the identified peptides belong to the pathways indicating the involvement of these proteins in extracellular processes, as “extracellular region part” (GO:0044421), “extracellular exosome” (GO:0070062) and “membrane-bounded vesicle” (GO:0031988). All significant pathways can be found in Supplementary Table S3.

## 2 Experimental design, materials and methods

### 2.1 Patients and specimens

Blood plasma and serum samples of 10 healthy male (average age 32 years) and 10 healthy female (average age 26 years) donors were collected from the Federal Research and Clinical Center of Physical Chemical Medicine of the Federal Medical and Biological Agency of the Russian Federation. The status of “healthy” was set based on anamnesis and further monitoring of the donor. The study was approved by the Ethics Committees of the clinical center and all the donors gave written informed consent for the participation in the study.

### 2.2 Sample collection

To obtain plasma, blood samples were collected from cubital vein into blood collection tubes (REF 456023, 6 ml, Vacuette tube, Austria). Plasma was obtained within 15 min after collection. The collection tubes were centrifuged for 15 min at 700 g at room temperature. The plasma was separated from blood cells, aliquoted and stored at –80 °C until analysis. To obtain serum, blood samples were collected from cubital vein into blood collection tubes (REF 456092, 6 ml, Vacuette tube, Austria). Serum was obtained after coagulation of blood for 1 h at room temperature. The collection tubes were centrifuged for 15 min at 700 g at room temperature. The serum was separated from the clot, aliquoted and stored at –80 °C until analysis.

### 2.3 Chemicals

Formic acid (FA), trifluoroacetic acid (TFA), acetic acid (AcOH), ammonium hydroxide solution (NH4OH) and sodium hydroxide (NaOH) were purchased from Sigma-Aldrich (St. Louis, MO, USA). LiChrosolv acetonitrile hypergrade for LC–MS (AсN), LiChrosolv acetone for liquid chromatography, LiChrosolv methanol gradient grade for liquid chromatography (MeOH), LiChrosolv ethanol gradient grade for liquid chromatography and HPLC grade water were acquired from Merck (Darmstadt, Germany).

### 2.4 Plasma and serum fractionation and peptide extraction

#### 2.4.1 Peptide extraction using cation exchange Toyopearl, Bio-Gel or SP-Sephadex

Blood plasma and serum samples were fractionated either on Toyopearl CM-650M (Tosoh Bioscience LLC, USA) weak cation exchange particles, or CM Bio-Gel A (Bio-Rad Laboratories, Inc., USA) cation exchange gel, or SP Sephadex C-25 (GE Healthcare Bio-Sciences AB, Sweden) strong cation exchange particles. Preliminarily, 80 µl of sorbent was washed 2 times with 400 µl WS1 (20 mM AcOH (pH 3.5), NaOH) in an eppendorf tube. To precipitate the sorbent, the eppendorf tube was centrifuged at 500 g for 10 s. Then the solvent was accurately removed paying close attention not to take any sorbent beads. 200 µl of plasma/serum was diluted with 400 µl WS1 and added to the sorbent. After 30 min of incubation with vortexing, the sorbent was precipitated and the plasma/serum-buffer solution was removed. Then the sorbent was washed 3 times with 700 µl WS1. The sorbent was incubated for 15 min with 800 µl of 0.1% NH4OH (pH 11), precipitated and the eluate was collected. Finally, 9 µl of FA was added to eluate to adjust pH to approximately 3.

#### 2.4.2 Peptide extraction using anion exchange QAE-Sephadex

Blood serum samples were fractionated on QAE Sephadex A-25 (GE Healthcare Bio-Sciences AB, Sweden) strong anion exchange particles. Preliminarily, 160 µl of sorbent was washed 2 times with 400 µl WS3 (20 mM Tris (pH 8.26), FA) in an eppendorf tude. To precipitate the sorbent, the eppendorf tude was centrifuged at 500 g for 10 s. Then the solvent was accurately removed paying close attention not to take any sorbent beads. 200 µl of serum was diluted with 400 µl WS3 and added to the sorbent. After 30 min of incubation with vortexing, the sorbent was precipitated and the serum-buffer solution was removed. Then the sorbent was washed 3 times with 700 µl WS3. The sorbent was incubated for 15 min with 800 µl of 0.5% TFA, precipitated and the eluate was collected.

#### 2.4.3 Peptide desorption from abundant blood proteins

To desorb peptides from the surface of abundant blood proteins, we used the technique described earlier [3]. The eppendorf with the eluate after peptide extraction was incubated at 98 °C water for 15 min. After heating, additional fractionation was carried out on a C18 Discovery Supelco 50 mg (Sigma-Aldrich Co. LLC, USA) RP-SPE cartridge. The RP-SPE cartridge was pre-conditioned with 500 µl of MeOH and equilibrated 3 times with 500 µl of WS5 (3% AcN, 97% water, 0.1% TFA). Eluate from the previous step was applied on the sorbent and went through at a flow rate of approximately 200 µl/min. The cartridge was washed 3 times with 300 µl WS5. Eluate was collected with 1 ml 80% AcN, 20% water, 0.1%TFA, concentrated under vacuum to 5 µl and diluted with 10 µl WS5.

### 2.5 LC–MS/MS analysis

Analysis was performed on a TripleTOF 5600+ mass spectrometer with a NanoSpray III ion source (Sciex, Canada) coupled with a NanoLC Ultra 2D+ nano-HPLC system (Eksigent, USA). The HPLC system was configured in the trap-elute mode. For sample loading buffer and buffer A, a mixture of 98.9% water, 1% MeOH, 0.1% FA (v/v) was used. Buffer B was 99.9% AcN and 0.1% FA (v/v). Samples were loaded on a Chrom XP C18 trap column (3 µm, 120 Å, 350 µm 0.5 mm; Eksigent) at a flow rate of 3 µl/min for 10 min and eluted through a 3C18-CL-120 separation column (3 µm, 120 Å, 75 µm 150 mm; Eksigent) at a flow rate of 300 nl/min. The gradient was from 5% to 40% buffer B in 90 min followed by 10 min at 95% buffer B and 20 min of re-equilibration with 5% buffer B. Between different samples, two blank 45-min runs consisting of 5–8 min waves (5% B, 95%, 95%, 5%) were required to wash the system and to prevent carryover. The information-dependent mass-spectrometer experiment included one survey MS1 scan followed by 50 dependent MS2 scans. MS1 acquisition parameters were as follows: the mass range for MS2 analysis was 300–1250 *m*/*z*, and the signal accumulation time was 250 ms. Ions for MS2 analysis were selected on the basis of intensity with a threshold of 200 counts per second and a charge state from 2 to 5. MS2 acquisition parameters were as follows: the resolution of the quadrupole was set to UNIT (0.7 Da), the measurement mass range was 200–1800 *m*/*z*, and the signal accumulation time was 50 ms for each parent ion. Collision-activated dissociation was performed with nitrogen gas with the collision energy ramped from 25 to 55 V within the signal accumulation time of 50 ms. Analyzed parent ions were sent to the dynamic exclusion list for 15 s in order to get an MS2 spectra at the chromatographic peak apex. β-Galactosidase tryptic solution (20 fmol) was run with a 15-min gradient (5–25% buffer B) every two samples and between sample sets to calibrate the mass spectrometer and to control the overall system performance, stability, and reproducibility.

### 2.6 Peptide identification

Raw LC–MS/MS data were converted to .mgf peaklists with ProteinPilot (version 4.5, Sciex, Canada). For this procedure, we ran ProteinPilot in identification mode with the following parameters: no specific digestion, TripleTOF 5600 instrument, thorough ID search with detected protein threshold 95.0% against the UniProt human protein knowledgebase. For thorough protein identification, the generated peak lists were searched with the MASCOT (version 2.5.1, Matrix Science Ltd., UK) and X! Tandem (VENGEANCE, 2015.12.15, The Global Proteome Machine Organization) search engines against the UniProt human protein knowledgebase. The precursor and fragment mass tolerance were set at 20 ppm and 50 ppm, respectively. Database-searching parameters included the following: no specific digestion. For X! Tandem we also selected parameters that allowed quick check for protein N-terminal residue acetylation, peptide N-terminal glutamine ammonia loss or peptide N-terminal glutamic acid water loss. Resulting files were submitted to the Scaffold 4 software (version 4.2.1, Proteome Software, Inc., USA) for validation and further analysis. We used the local false discovery rate scoring algorithm with standard experiment-wide protein grouping. For the evaluation of peptide hits, a false discovery rate less than 1% was selected for peptides only. False positive identifications were based on reverse database analysis.

### 2.7 Protein–protein interaction network and enrichment analysis

Precursor proteins, which peptides were identified in at least two samples (one sample of a male donor and one sample of a female donor), were analysed via STRING (Search Tool for the Retrieval of Interacting Genes/Proteins) website [6]. The list of the precursor protein identifies were uploaded and standard enrichment was performed using Gene Ontology (GO): Biological Process, Molecular Function or Cellular Component; Kyoto Encyclopedia of Genes and Genomes (KEGG) Pathways; Protein Families (PFAM) Protein Domains. False discovery rate threshold was 0.05.
